# Illness Characteristics of COVID-19 in Children Infected with the SARS-CoV-2 Delta Variant

**DOI:** 10.3390/children9050652

**Published:** 2022-05-03

**Authors:** Erika Molteni, Carole H. Sudre, Liane Dos Santos Canas, Sunil S. Bhopal, Robert C. Hughes, Liyuan Chen, Jie Deng, Benjamin Murray, Eric Kerfoot, Michela Antonelli, Mark Graham, Kerstin Kläser, Anna May, Christina Hu, Joan Capdevila Pujol, Jonathan Wolf, Alexander Hammers, Timothy D. Spector, Sebastien Ourselin, Marc Modat, Claire J. Steves, Michael Absoud, Emma L. Duncan

**Affiliations:** 1 School of Biomedical Engineering & Imaging Sciences, King’s College London, London WC2R 2LS, UK; erika.molteni@kcl.ac.uk (E.M.); carole.sudre@kcl.ac.uk (C.H.S.); liane.dos_santos_canas@kcl.ac.uk (L.D.S.C.); liyuan.chen@kcl.ac.uk (L.C.); jie.1.deng@kcl.ac.uk (J.D.); benjamin.murray@kcl.ac.uk (B.M.); eric.kerfoot@kcl.ac.uk (E.K.); michela.antonelli@kcl.ac.uk (M.A.); mark.graham@kcl.ac.uk (M.G.); kerstin.klaser@kcl.ac.uk (K.K.); alexander.hammers@kcl.ac.uk (A.H.); sebastien.ourselin@kcl.ac.uk (S.O.); marc.modat@kcl.ac.uk (M.M.); 2 MRC Unit for Lifelong Health and Ageing, Department of Population Health Sciences, University College London, London WC1E 6BT, UK; 3 Centre for Medical Image Computing, Department of Computer Science, University College London, London WC1E 6BT, UK; 4 Population Health Sciences Institute, Faculty of Medical Sciences, Newcastle University, Newcastle upon Tyne NE1 7RU, UK; sunil.bhopal@newcastle.ac.uk; 5 Department of Population Health, Faculty of Epidemiology & Population Health, London School of Hygiene & Tropical Medicine, Keppel Street, London WC1E 7HT, UK; robert.hughes@lshtm.ac.uk; 6 ZOE Limited London, London SE1 7RW, UK; anna.may@joinzoe.com (A.M.); christina@joinzoe.com (C.H.); joan@joinzoe.com (J.C.P.); jonathan@joinzoe.com (J.W.); 7 King’s College London & Guy’s and St Thomas’ PET Centre, London WC2R 2LS, UK; 8 Department of Twin Research and Genetic Epidemiology, King’s College London, London WC2R 2LS, UK; tim.spector@kcl.ac.uk (T.D.S.); claire.j.steves@kcl.ac.uk (C.J.S.); 9 Department of Aging and Health, Guy’s and St Thomas’ NHS Foundation Trust, London SE1 7EH, UK; 10 Children’s Neurosciences, Evelina London Children’s Hospital, St Thomas’ Hospital, King’s Health Partners, Academic Health Science Centre, London SE1 7EH, UK; 11 Department of Women and Children’s Health, Faculty of Life Sciences and Medicine, School of Life Course Sciences, King’s College London, London WC2R 2LS, UK; 12 Department of Endocrinology, Guy’s and St Thomas’ NHS Foundation Trust, London SE1 7EH, UK

**Keywords:** SARS-CoV-2 Delta strain, paediatric COVID-19, COVID-19 symptoms, SARS-CoV-2 B.1.617.2 variant, SARS-CoV-2 B.1.1.7 variant

## Abstract

Background: The Delta (B.1.617.2) SARS-CoV-2 variant was the predominant UK circulating strain between May and November 2021. We investigated whether COVID-19 from Delta infection differed from infection with previous variants in children. Methods: Through the prospective COVID Symptom Study, 109,626 UK school-aged children were proxy-reported between 28 December 2020 and 8 July 2021. We selected all symptomatic children who tested positive for SARS-CoV-2 and were proxy-reported at least weekly, within two timeframes: 28 December 2020 to 6 May 2021 (Alpha (B.1.1.7), the main UK circulating variant) and 26 May to 8 July 2021 (Delta, the main UK circulating variant), with all children unvaccinated (as per national policy at the time). We assessed illness profiles (symptom prevalence, duration, and burden), hospital presentation, and presence of long (≥28 day) illness, and calculated odds ratios for symptoms presenting within the first 28 days of illness. Results: 694 (276 younger (5–11 years), 418 older (12–17 years)) symptomatic children tested positive for SARS-CoV-2 with Alpha infection and 706 (227 younger and 479 older) children with Delta infection. Median illness duration was short with either variant (overall cohort: 5 days (IQR 2–9.75) with Alpha, 5 days (IQR 2–9) with Delta). The seven most prevalent symptoms were common to both variants. Symptom burden over the first 28 days was slightly greater with Delta compared with Alpha infection (in younger children, 3 (IQR 2–5) symptoms with Alpha, 4 (IQR 2–7) with Delta; in older children, 5 (IQR 3–8) symptoms with Alpha, 6 (IQR 3–9) with Delta infection ). The odds of presenting several symptoms were higher with Delta than Alpha infection, including headache and fever. Few children presented to hospital, and long illness duration was uncommon, with either variant. Conclusions: COVID-19 in UK school-aged children due to SARS-CoV-2 Delta strain B.1.617.2 resembles illness due to the Alpha variant B.1.1.7., with short duration and similar symptom burden.

## 1. Introduction

Viruses acquire genetic changes over time [1], which can alter transmissibility [2], virulence [3], clinical presentation [4,5], and/or effectiveness of vaccination and other health measures [6]. Correspondingly, multiple SARS-CoV-2 variants have emerged during the pandemic, with several variants of concern circulating internationally as a result (https://www.who.int/en/activities/tracking-SARS-CoV-2-variants/ accessed on 2 May 2022). The Alpha variant (B.1.1.7) initially emerged in the UK in September 2020 (https://www.who.int/en/activities/tracking-SARS-CoV-2-variants/ accessed on 2 May 2022), and was the predominant UK strain from November 2020 until early May 2021 (https://assets.publishing.service.gov.uk/government/uploads/system/uploads/attachment_data/file/959438/Technical_Briefing_VOC_SH_NJL2_SH2.pdf; https://assets.publishing.service.gov.uk/government/uploads/system/uploads/attachment_data/file/1014926/Technical_Briefing_22_21_09_02.pdf; both accessed on 2 May 2022). The Delta variant (B.1.617.2) was initially identified in India in October 2020, with the Delta lineage designated a “variant of concern” by WHO on 11 May 2021 (https://www.who.int/en/activities/tracking-SARS-CoV-2-variants/ accessed on 2 May 2022). By the end of May 2021, the Delta variant was the predominant (>75%) circulating variant in the UK, reaching >99% in the week commencing on June 27, 2021, and remaining predominant until November 2021 (Supplementary Table S1: https://assets.publishing.service.gov.uk/government/uploads/system/uploads/attachment_data/file/1018547/Technical_Briefing_23_21_09_16.pdf accessed on 2 May 2022). The Delta variant appeared more transmissible than wild-type and previous variants, with transmissibility approximately 60% higher than the Alpha variant [7,8]. There was some evidence that COVID-19 due to infection with the Delta variant might be more severe than illness caused by previous strains, with increased requirements for hospitalisation and respiratory support [9,10]; in addition, available vaccines might have been modestly less efficacious against the Delta variant than preceding variants, although still very effective against severe illness and death [11].

In adults, comparisons of COVID-19 caused by the Delta variant vs. other variants is particularly complicated by vaccination roll-out and altered clinical presentation in individuals infected post-vaccination [12]. However, children in the UK were not offered vaccinations until after Delta became the main circulating variant. The BNT162b2 vaccine (Comirnaty, Pfizer) was ultimately authorized for SARS-CoV-2 immunization in minors in the UK, with roll-out as follows: at-risk minors (i.e., with severe neuro-disabilities, Down’s syndrome, immunosuppression, multiple or severe learning disabilities) aged ≥12 years, and those co-living with immunosuppressed individuals, were offered vaccination after July 19, 2021; children aged 16–17 years (single dose vaccination) after August 4, 2021; children aged ≥12 years (single dose vaccination) after 13 September 2021 (https://www.gov.uk/government/news/jcvi-issues-advice-on-covid-19-vaccination-of-children-and-young-people accessed on 2 May 2022).

Here, we describe and compare illness profiles in symptomatic UK school-aged children with SARS-CoV-2 infection presenting during two timeframes: 28 December 2020 (the peak of the 2020/21 UK winter wave) to 6 May 2021, when the Alpha variant was the main UK circulating variant, and 26 May 2021 to 8 July 2021, when Delta was the predominant variant (https://www.gov.uk/government/publications/covid-19-variants-genomically-confirmed-case-numbers accessed on 2 May 2022), noting that, during these timeframes, the vaccination campaign did not include UK school-aged children. Information on the evolving presentation of symptoms related to SARS-CoV-2 variants is not just of academic interest: it informs the changing burden for healthcare services.

## 2. Materials and Methods

### 2.1. Study and Public Involvement

This study used data from the COVID Symptom Study, obtained via the ZOE COVID Study App (details previously published in [13]). Briefly, this prospective study collects self-reported data from adult participants through a mobile application (app), including direct questions about specific symptoms (Supplementary Table S2), free text symptom reporting, SARS-CoV-2 testing, vaccination, and healthcare access, as well as demographic and co-morbidity data. Regular daily reporting is encouraged by prompting alerts. Adult contributors can proxy-report on behalf of children; however, data cannot be linked between the contributor and the proxy-reported individual. Children aged 16–17 years can also self-report. Questions about symptoms and co-morbidities were largely informed by adult data [14], and do not include some paediatric co-morbidities (e.g., neurological or neuro-disability disorders). Ethics approval for this study was granted by the KCL Ethics Committee (LRS-19/20-18210). Upon registration through the app, all participants provided consent for their data to be used for COVID-19 research. Permission was specifically granted to enable use of proxy-reported data, including data from minors.

### 2.2. Participants

Data from all proxy-reported UK school-aged children (5–17 years) with a positive test between 28 December 2020 to 8 July 2021 (i.e., prior to vaccination approval for any child in the UK) were selected. As previously [15], children were considered to have COVID-19 if proxy-reported with relevant symptoms between 2 weeks before and 1 week after SARS-CoV-2 infection confirmation (either PCR or LFAT). Data were included for children proxy-reported at least once weekly from first symptom report until healthy, or until proxy-reporting ceased, noting that our previous study showed proxy-reporting in the included cohort to be assiduous (continued logging until healthy in >90% of children) [13].

As a proxy for other circulating viruses and possible co-infection influencing illness profile, we considered symptoms in children who tested negative for SARS-CoV-2 within the same timeframes, and provide context with Public Health England data for circulating viruses other than COVID-19 (Public Health England DataMart estimates based on sentinel laboratory surveillance; https://assets.publishing.service.gov.uk/government/uploads/system/uploads/attachment_data/file/1016276/Weekly_Flu_and_COVID-19_report_w36.pdf Figure 16 accessed on 2 May 2022). As previously [13], self-reported data from children aged 16–17 years were excluded due to lower assiduity in reporting, and to avoid potential data duplication (i.e., both proxy-reporting and self-reporting).

### 2.3. Data Analysis

Illness characteristics were assessed for children logging a positive test within two timeframes, according to whether Alpha or Delta was the predominant circulating UK SARS-CoV-2 variant at the time (Supplementary Table S1). The first was from 28 December 2020 to 6 May 2021 (noting 29 December 2020 was the peak positive specimen date for the 2020–2021 UK winter wave of the pandemic, https://assets.publishing.service.gov.uk/government/uploads/system/uploads/attachment_data/file/975754/Variants_of_Concern_Technical_Briefing_8_Data_England.xlsx accessed on 2 May 2022). The second was from 26 May 2021 to 8 July 2021 (https://assets.publishing.service.gov.uk/government/uploads/system/uploads/attachment_data/file/959359/Variant_of_Concern_VOC_202012_01_Technical_Briefing_4.pdf AND https://assets.publishing.service.gov.uk/government/uploads/system/uploads/attachment_data/file/1018476/Variants_of_Concern_Technical_Briefing_23__Data_England.xlsx AND https://assets.publishing.service.gov.uk/government/uploads/system/uploads/attachment_data/file/1018547/Technical_Briefing_23_21_09_16.pdf all accessed on 2 May 2022). Infection presenting in the first timeframe was assumed to be due to the Alpha variant, and in the second to the Delta variant; terminology herein reflects these assumptions. However, individual test results regarding variant strain were not available. Data were analysed overall, and within two age groups: younger children aged 5–11 years (UK primary school-aged children) and older children aged 12–17 years (UK secondary school-aged children, noting that children can leave school after the age of 16 years, and rate of leaving is ≤6% in the UK) (https://assets.publishing.service.gov.uk/government/uploads/system/uploads/attachment_data/file/860135/Destinations_main_text_2020_REV.pdf accessed on 2 May 2022).

Illness duration was calculated from first symptom (having been previously asymptomatic) until recovery (return to asymptomatic or, if proxy-reporting ceased before becoming asymptomatic, final proxy report). Individuals proxy-reported as asymptomatic but subsequently re-reported with symptoms within 1 week of their last symptomatic report were considered unwell from initial presentation (i.e., having relapsing or remitting illness), and the illness duration was calculated accordingly. Individual symptom prevalence was assessed according to the duration between the first and last report for that symptom. Participant selection was determined by the date of positive SARS-CoV-2 testing; however, symptoms were considered over the entire illness duration, which could extend outside the test date boundaries to a maximum of 2 weeks before (by virtue of the definition of COVID-19 for this study) and 4 weeks after the timeframe boundaries (by virtue of data censoring). Data were censored on 5 August 2021, 28 days after the last date of inclusion for children whose illness commenced during the Delta period; thus, the duration of individual symptoms and of overall illness for children who were ill for >28 days could not be robustly determined for every child. Instead, we report the number of children who were still unwell at 28 days for both timeframes, and otherwise exclude them from descriptive statistics regarding symptom and illness duration. Importantly, our previous study of COVID-19 in children did not demonstrate new symptoms appearing later than 28 days after a positive test [13]; for the current study, however, we scrutinised data from children with long illness durations for new symptoms presenting after Day 28. Odds ratios were calculated to compare symptom presentation with Delta vs. Alpha infection, considering all symptoms presenting within 28 days. Symptom burden (number of symptoms) was calculated over two timeframes: within the first week (<7 days) and over the entire course of illness (considered to be 28 days). Symptom burden was considered for all symptoms, and with respect to the seven most common symptoms for each variant, which seven symptoms proved common to both cohorts (considered across the entire age range). Children presenting to hospital (comprising presentation to the emergency department or admission to hospital) for each timeframe were also counted.

### 2.4. Statistical Analyses

Data are presented as descriptive statistics. Tests for differences in counts and raw prevalence were not conducted, given the unmatched nature of the cohorts and the many differences between the two timeframes (including school opening, social distancing regulations, and changing seasons). Where robust comparisons were possible (e.g., demographic data), Wilcoxon signed-rank testing, two-tailed χ² tests, or Fisher’s exact tests were used. Odds ratios were calculated using logistic regression for all symptoms, unless having null prevalence, with age and gender as covariates. Body mass index was not used, as its robustness and utility are debated for paediatric cohorts. False discovery rate (FDR) correction was applied to multiple tests on non-independent data to adjust the level of statistical significance [16]. All analyses were performed in Python version 3.7.

## 3. Results

**Figure 1:**
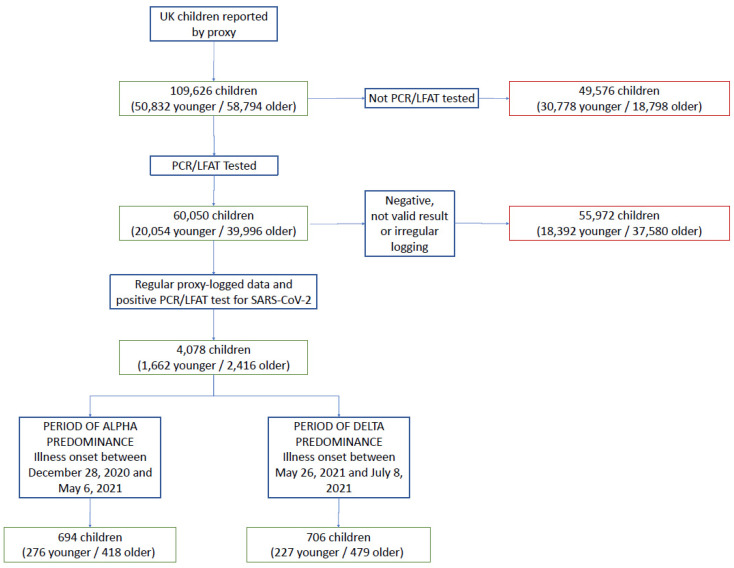
Study flowchart of inclusion and exclusion criteria. Overall number for the entire cohort of children is given first. Younger children = aged 5–11 years (UK primary school-aged children). Older children = aged 12–17 years (UK secondary school-aged children). Not valid result = test result proxy-reported as “failed test” or “still waiting”. Irregular logging = proxy-reporting with intervals of more than 7 days between proxy-reports during illness.

Overall, 109,626 UK children aged 5–17 years (50,832 younger and 58,794 older children) were proxy-reported between 28 December 2020 and 8 July 2021. Of these, 60,050 (20,054 younger and 39,996 older children) were tested for SARS-CoV-2 (Figure 1). We considered symptomatic children testing positive for SARS-CoV-2, with sufficient proxy-logging for illness profiling. A total of 694 children (276 younger, 418 older children) tested positive between 28 December 2020 and 6 May 2021, assumed to be with the Alpha variant; 706 children (227 younger, 479 older children) tested positive between 26 May 2021 and 8 July 2021 (with symptom onset on or before 8 July in all children), assumed to be with the Delta variant (Table 1).

Median illness duration with COVID-19 in the cohort overall was 5 days (IQR 2–9.75) with Alpha and 5 days (IQR 2–9) with Delta infection. Illness duration in younger children was 4 (IQR 2–8) days with Alpha and 4 (IQR 2–7.5) days with Delta infection, and in older children, 6 (IQR 3–10) days with Alpha and 6 (IQR 3–10) days with Delta infection. Although illness duration appeared slightly shorter in younger children than older children within each variant’s time period, this was not significant (younger children vs. older children during the Alpha period Z= 0.53, *p* = 0.594, and during the Delta period Z = 0.74, *p* = 0.458).

**Figure 2:**
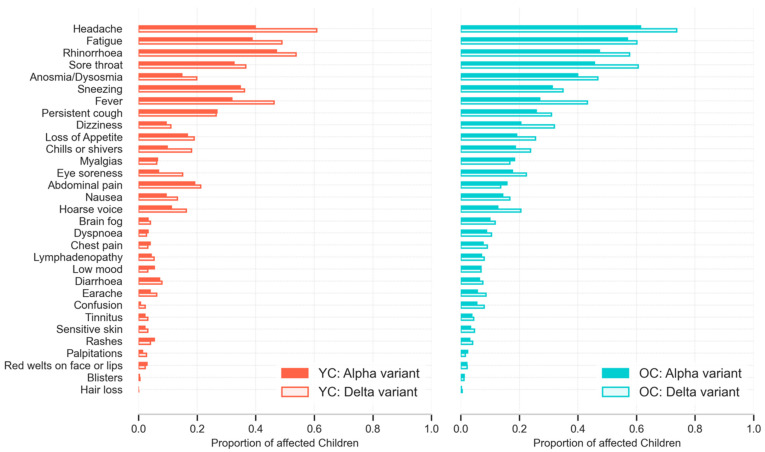
Prevalence of symptoms reported over the course of illness (up to 28 days) in younger (YC, 5–11 years) and older (OC, 12–17 years) children with COVID-19 during periods of SARS-CoV-2 Alpha or Delta variant predominance.

Individual symptom prevalence in children with Alpha or Delta infection are presented in Figure 2 and Supplementary Table S3. In younger children, the most common symptoms were the same with either variant: rhinorrhoea (“runny nose”) (130 (47.1%) of 276), headache (110 (39.9%) of 276), and fatigue (107 (38.8%) of 276) with Alpha infection; headache (138 (60.8%) of 227), rhinorrhoea (122 (53.7%) of 227), and fatigue (111 (48.9%) of 227) with Delta infection. In older children with Alpha infection, the three most common symptoms were, again, headache (257 (61.5%) of 418), fatigue (238 (56.9%) of 418), and rhinorrhoea (198 (47.4%) of 418); in contrast, for Delta they were headache (353 (73.7%) of 479), sore throat (290 (60.5%) of 479), and fatigue (288 (60.1%) of 479). The top seven symptoms for each cohort were common to both variants: headache, fatigue, fever, dysosmia (encompassing both anosmia and dysosmia), sneezing, rhinorrhoea, and sore throat, although there were some differences in prevalence order (Figure 2).

**Figure 3:**
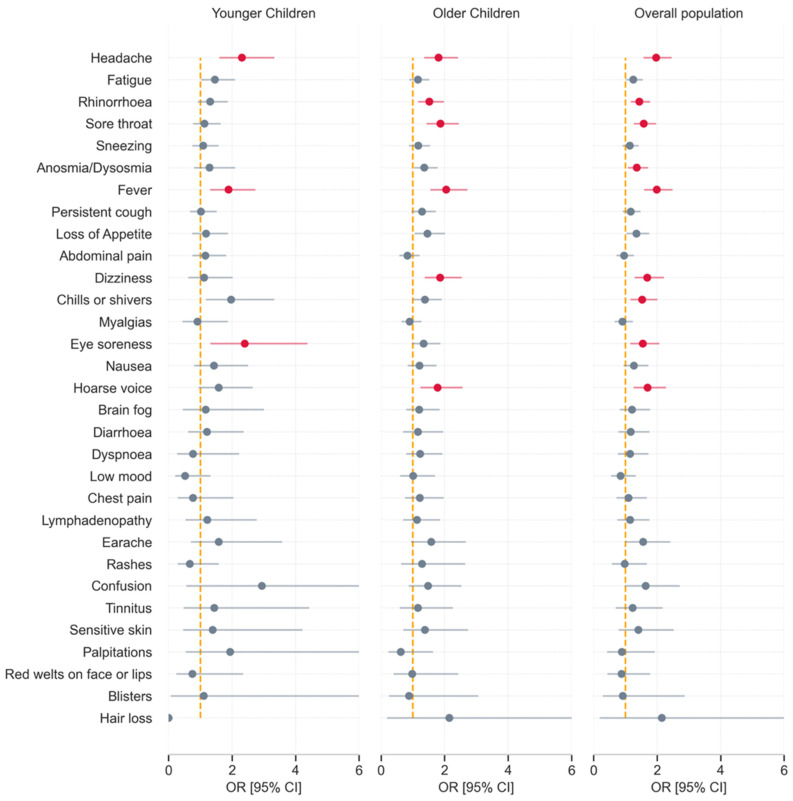
Odds ratios for a symptom presenting within the first 28 days of illness in children with COVID-19 during periods of SARS-CoV-2 Delta vs. Alpha variant predominance. Results for younger (5–11 years) and older (12–17 years) children, and for the cohort overall, comparing symptom prevalence during Delta with prevalence during Alpha infection, and adjusted for age and sex. Results with statistical significance after false discovery rate test (α < 0·05) are in red.

Odds ratios were computed for any symptom presenting by day 28, comparing Delta to Alpha infection (Figure 3, Supplementary Table S4). Significance was determined after FDR correction, thus some odds ratios that do not cross the unit value are not significant. Considered overall, several symptoms were more common with Delta than with Alpha infection: headache, rhinorrhoea, sore throat, dysosmia, fever, dizziness, “chills or shivers”, eye soreness, and hoarse voice. Considered within age groups, six symptoms were more common with Delta compared with Alpha infection in older children, and three in younger children (Figure 3, in red).

Median symptom burden, considered over the entire illness and for the overall cohort, was 4 (IQR 2–7) during the Alpha period, and 5 (IQR 3–8) during Delta. In more detail, it was 3 (IQR 2–5) symptoms with Alpha and 4 (IQR 2–7) with Delta infection in younger children, and 5 (IQR 3–8) symptoms with Alpha and 6 (IQR 3–9) with Delta infection in older children. Considering only the symptoms experienced in the first week of illness, and for the overall cohort, median symptom burden was 4 (IQR 2–6) with Alpha and 5 (IQR 3–8) with Delta. Median symptom burden was 3 (IQR 2–5) with Alpha and 4 (IQR 2–6) with Delta infection in younger children, and 5 (IQR 3–7) with Alpha and 6 (IQR 3–9) with Delta infection in older children, noting here that median illness duration was less than a week with either variant and in both age groups. (Table 1). Considering the seven most common symptoms (as above, common to both variants), the median burden was 2 (IQR 1–3) with Alpha and 3 (IQR 2–4) with Delta infection in younger children, and 3 (IQR 2–4) with Alpha and 4 (IQR 2–5) with Delta in older children. 

**Figure 4:**
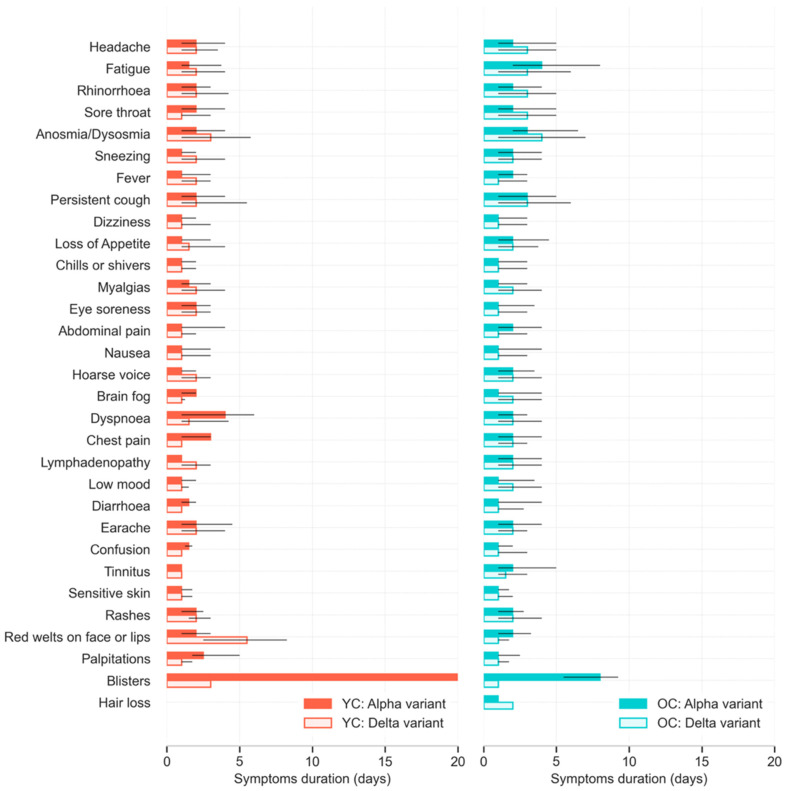
Median duration and IQR of each symptom reported over the course of illness in younger (5–11 years) and older (12–17 years) children with COVID-19, whose illness lasted <28 days, during periods of SARS-CoV-2 Alpha or Delta variant predominance.

Individual symptom duration was short (median 5 days) (Figure 4). The symptom of longest duration was ‘blisters’ (particularly in younger children with Alpha infection); however, the prevalence of blisters was extremely low (approx. 1%) (Supplementary Table S3).

Presentation to hospital was reported for 14 younger and 16 older children: 6 [2.2%] of 276 younger and 8 [1.9%] of 418 older children with Alpha infection, and 8 [3.5%] of 227 younger and 8 [1.7%] of 479 older children with Delta infection (Table 1). No deaths were reported. Illness longer than 28 days was reported for 5 younger and 22 older children overall (Table 1). Illness had resolved by 28 days in 274 (99.3%) of 276 younger, and in 408 (97.6%) of 418 older children with Alpha infection, and in 224 (98.7%) of 227 younger, and in 467 (97.5%) of 479 older children with Delta infection. For those children with longer illness duration, no new symptoms developed after day 28. 

Symptoms in children testing negative for SARS-CoV-2 during the same Alpha and Delta timeframes are presented in Supplementary Table S5 and Supplementary Figure S1. Rhinorrhoea, sore throat, sneezing, and persistent cough were more common during the Delta period, and headache and fatigue during the Alpha period (not statistically tested).

## 4. Discussion

Here we present the illness profile of COVID-19 in UK school-aged children with symptomatic SARS-CoV-2 Delta (B.1.617.2) variant infection, and compare this with similar data for symptomatic children with SARS-CoV-2 Alpha (B.1.1.7) variant infection. Several individual symptoms were more common with Delta infection (particularly in older children), and symptom burden appeared slightly greater, in both age groups. However, most symptoms were of short duration (median <2 days); overall illness duration was short (median 5 days); few children required hospital attention; and illness lasting for more than 28 days was uncommon. Our data suggest that the clinical characteristics of COVID-19 due to the Delta variant in children are broadly similar to those associated with other COVID-19 variants [13].

For both variants, the symptom with longest duration was ‘blisters’, although this symptom was rare. Five types of cutaneous lesions have been identified with COVID-19 [17], of which pseudo-chilblains, mainly reported in children, are at the lowest spectrum of severity; these resolve spontaneously, albeit slowly, in most cases. As children were not clinically examined, nor photos submitted, we are not able to comment further on the nature of these lesions.

To our knowledge, this is the first large-scale epidemiological study comparing illness characteristics of children with Alpha vs. Delta infection. The CloCk matched cohort study reported recently on a UK cohort tested from January to March 2021, which was during Alpha variant predominance (https://www.researchsquare.com/article/rs-798316/v1 accessed on 2 May 2022). In addition, the UK Office for National Statistics (ONS) has examined the prevalence of prolonged symptoms in children at several timepoints across the pandemic; however, to date, no comparison of symptoms between timepoints has been presented (https://www.ons.gov.uk/peoplepopulationandcommunity/healthandsocialcare/conditionsanddiseases/bulletins/prevalenceofongoingsymptomsfollowingcoronaviruscovid19infectionintheuk/2september2021 accessed on 2 May 2022). Whilst some cohort studies have included comparisons between variants in adult and child populations considered jointly, child-specific data have not been reported [9]. The latest report from the USA Centers for Disease Control indicates that differences in disease severity and duration between Delta and previous variants in children are unclear (https://www.cdc.gov/mmwr/volumes/70/wr/mm7036e1.htm?s_cid=mm7036e1_w accessed on 2 May 2022). However, although USA cases in children have increased recently, the proportion of hospitalised children requiring intensive care is unchanged, suggesting that infection severity is, at least, no worse (and possibly less) with the evolution of circulating variants [18]. In England [9] and Scotland [19], observational data from the population as a whole (no child-specific aggregation) suggest that hospitalisation may be higher with Delta compared to Alpha infection (noting that vaccination has changed the profile of illness in adults, and currently the majority of hospitalised adults in the UK are unvaccinated). In contrast, data from Norway, again with no separate analysis for children, do not support higher rates of hospitalization with the Delta variant [20]. Overall, the numbers of presentations to hospital in our study participants appear lower than independent estimates [21]; however, the SARS-CoV-2 testing policy and hospital admission policy may differ between studies, limiting comparability.

With the exception of odds ratios for symptom presence, which could be robustly tested, our data are descriptive. We included data from all regularly proxy-logged children with a positive test during the two timeframes, noting the many differences between the two groups that could not be controlled for. As for many children worldwide, the pandemic has been extremely disruptive for UK schooling. Lockdown was re-imposed across the UK just before Christmas 2020, and primary and secondary schools were closed throughout January and February 2021. Schools re-opened from March 8 until the end of the school year (nominally, end of July) (https://www.gov.uk/government/publications/covid-19-response-spring-2021 accessed on 2 May 2022). However, in-person school attendance continued to be compromised on an ad hoc and intermittent basis for many children (>2% of children in the UK, for example, were at-home quarantining whilst awaiting a test result, whether for the child personally, a family member, or classmates). Differences in school attendance may have affected our data in multiple ways, including differing daily routines (e.g., linking school attendance with proxy-reporting habits), exposure to SARS-CoV-2 at school and on public transport, and testing of children according to parental decisions regarding school attendance.

The UK national lockdown was progressively lifted after March 29, 2021, which altered the prevalence of SARS-CoV-2 and other circulating respiratory viruses. Rhinovirus prevalence, which had increased rapidly at the start of the school year in September 2020, suddenly fell in January 2021, only to rise again from March 2021 (concordant with school re-opening) (https://assets.publishing.service.gov.uk/government/uploads/system/uploads/attachment_data/file/1016276/Weekly_Flu_and_COVID-19_report_w36.pdf Figure 16 accessed on 2 May 2022). Other tested viruses (adenovirus, human metapneumovirus, respiratory syncytial virus, and parainfluenza) were at unusually low levels during January to April 2021. With the lifting of lockdown, a rapid and unseasonal increase in parainfluenza prevalence was observed, followed by a rise in respiratory syncytial virus to unusually high levels (which continues to be high at time of writing). Thus, we considered whether our data might inadvertently capture symptoms from viruses other than COVID-19. As all children included in the Alpha and Delta COVID-19 groups had a positive SARS-CoV-2 test, this could indicate co-infection. Although we could not formally test this, we have included illness profiles from symptomatic children testing negative for SARS-CoV-2 during the two timeframes; rhinorrhoea, sore throat, sneezing, and persistent cough were more common during the Delta period, with other symptoms being less common. However, the commonality of the top seven symptoms with either SARS-CoV-2 variant, and the other similarities in illness profiles, as detailed above, argue against co-infection; nevertheless, without simultaneous testing for other circulating viruses, the possibility of co-infection cannot be determined.

There were also large seasonal differences between the two timeframes (mid-winter to mid-spring vs. early-to-mid-summer), and, associated with this, varying prevalence of seasonal allergic rhinitis.

Testing policy also changed over time. At the start of this study, access to SARS-CoV-2 testing was symptom-based (informed by data from adults) [14], and required the presence of persistent cough, fever, and/or anosmia/dysosmia (https://www.nhs.uk/conditions/coronavirus-covid-19/symptoms/main-symptoms/ accessed on 2 May 2022). However, many other symptoms are manifest in infected individuals (both adults and children) [13,15,22], with headache and fatigue the most common symptoms in children [13,23]. Consistent with our previous work [24], and that of others [25], the current study supports the inclusion of other symptoms, particularly headache, if testing access for children is gated by symptom manifestation. Of note, an aim of regular (twice-weekly) and rapid testing of asymptomatic teachers and secondary school children was applied upon school re-opening.

Few individuals in our study were hospitalised; none died. A recent work analysing national databases reported no deaths in the UK in children aged 15 years or younger from either the Alpha or Delta variant [26]. Considering children aged 16–17 years, our database did not include any deaths in this age range; however, we acknowledge that in the remote case of a very dramatic event, such as the minor’s death, it is unlikely that the grieving relatives would take the time and effort to report their tragic loss to a phone app.

We recognize other weaknesses of our study. To begin, our study only assesses illness profiles in symptomatic children. We are therefore not able to comment on relative transmissibility, or on the prevalence of asymptomatic infection with Alpha vs. Delta variants. Furthermore, we only present data for symptoms assessed by direct questioning through the app across both timeframes (Supplementary Table S1), and any novel symptoms unique to the Delta variant would not be captured by our study. Our current study does not present illness profiles in children with illness for more than 28 days, to avoid bias introduced by our census timing; however, we do report the numbers of children with long illness durations, which were low with either variant. Our previous study showed that prolonged illness duration in children with COVID-19 is uncommon [13], consistent with the most recent (16 September 2021) publication of the UK ONS Coronavirus Infection Survey, which states that 3.3% (95%CI 2.5–4.5) of children aged 2–11 years testing positive for SARS-CoV-2 had 1 of 12 symptoms 29–56 days following infection, compared to 3.6% (95%CI 2.7–4.8) in a matched negative control group (note that this latter study may be more representative of children in the general population than our own [27], and includes children with asymptomatic infection) (https://www.ons.gov.uk/peoplepopulationandcommunity/healthandsocialcare/conditionsanddiseases/articles/technicalarticleupdatedestimatesoftheprevalenceofpostacutesymptomsamongpeoplewithcoronaviruscovid19intheuk/26april2020to1august2021 accessed on 2 May 2022). We did not ask any direct questions about multisystem inflammatory syndrome in children, noting the rarity of this severe post-infection condition. Lastly, and crucially, proxy-reported children depended on an adult participating in this app-based initiative who was willing and able to report through a smartphone app. COVID Symptom Study app users are predominantly female, had prevalently White ancestry, were older, and had an IMD status slightly higher than the UK average. Thus, children sampled in this study reflected this self-selection. Reporter awareness of COVID-19 symptoms may also have changed over time, which may have influenced their proxy-reporting for children.

## 5. Conclusions

In conclusion, our data show that COVID-19 due to Delta variant B.1.617.2 in UK school-aged children is usually of short duration and low symptom burden, similar to COVID-19 due to the Alpha variant B.1.1.7., with the seven most prevalent symptoms common to both strains. Few proxy-reported children presented to hospital, and the numbers of children with illness >28 days were low. Our study adds quantitative information around the manifestation of infection due to the Delta variant in children. More importantly, it provides a framework for the comparison of COVID-19 presentation in children due to emerging variants of the fast-evolving SARS-CoV-2 virus.

## Figures and Tables

**Table 1:** Characteristics of UK school-aged children presenting with COVID-19 during periods of SARS-CoV-2 Alpha or Delta variant predominance.

	Overall Symptomatic Children with Positive SARS-CoV-2 Test					
	Alpha Variant Period			Delta Variant Period		
	Younger Children (Aged 5–11 Years)	Older Children (Aged 12–17 Years)	Overall Cohort (Aged 5–17 Years)	Younger Children (Aged 5–11 Years)	Older Children (Aged 12–17 Years)	Overall Cohort (Aged 5–17 Years)
Number	276	418	694	227	479	706
Males (n (%))	144 (52.2)	213 (50.9)	357 (51.4)	102 (44.9)	255 (53.2)	357 (50.6)
Age, years (median (IQR))	9 (7–10)	15 (13–16)	12 (9–15)	9 (8–11)	15 (13–16)	14 (13–16)
BMI (kg/m^2^) (median (IQR))	16.44 (14.79–18.90)	19.53 (17.24–22.07)	18.16 (15.97–21.16)	16.68 (14.61–19.27)	18.97 (16.88–21.31)	18.21 (16.01–20.96)
Asthma (n (%))	32 (11.6)	49 (11.7)	81 (11.6)	24 (10.6)	56 (11.7)	80 (11.3)
Heart disease (n (%))	0 (0.0)	3 (0.9)	3 (0.4)	2 (0.7)	1 (0.2)	3 (0.4)
Diabetes (n (%))	1 (0.4)	4 (1.0)	5 (0.8)	1 (0.4)	1 (0.2)	2 (0.3)
Renal disease (n (%))	0 (0.0)	0 (0.0)	0 (0.0)	0 (0.0)	1 (0.2)	4 (0.1)
Number of children presenting to hospital * (n (%))	6 (2.2)	8 (1.9)	14 (2.0)	8 (3.5)	8 (1.7)	16 (2.2)
Illness duration (median (IQR))	4 (2–8)	6 (3–10)	5 (2–9.75)	4 (2–7.5)	6 (3–10)	5 (2–9)
Number of symptoms in the first week (median (IQR))	3 (2–5)	5 (3–7)	4 (2–6)	4 (2–6)	6 (3–9)	5 (3–8)
Number of children with illness duration ≥ 28 days (n (%))	2 (0.7)	10 (2.4)	14 (1.7)	3 (1.3)	12 (2.5)	15 (2.1)

BMI, Body Mass Index; IQR, Interquartile Range. * Presentation to hospital: presenting to the emergency department and/or admission to hospital.

## Data Availability

Data collected in the ZOE COVID Study App can be shared with other health researchers through the UK National Health Service-funded Health Data Research UK and Secure Anonymised Information Linkage consortium, housed in the UK Secure Research Platform (Swansea, UK). Anonymised data are available to be shared with researchers according to their protocols in the public interest https://web.www.healthdatagateway.org/dataset/594cfe55-96e3-45ff-874c-2c0006eeb881 accessed on 2 May 2022.

## Supplementary Materials

The following supporting information can be downloaded at: https://www.mdpi.com/article/10.3390/children9050652/s1. Figure S1. Prevalence of symptoms reported by children testing negative for COVID-19; Table S1. Incidence of the Alpha and Delta variants of SARS-CoV-2 in the United Kingdom; Table S2. List of symptom questions asked by the ZOE COVID Study app during the study period; Table S3. Symptoms over the entire illness duration in children testing positive for Alpha and Delta variants of SARS-CoV-2; Table S4. Odds ratios for symptoms presenting within 28 days of SARS-CoV-2 infection, comparing Delta to Alpha infection; Table S5. Symptom presentation over the first 28 days of the disease in children testing negative for SARS-CoV-2.

## Abbreviations

CI	Confidence interval
COVID-19	Coronavirus disease 2019
FDR	False discovery rate
IQR	Interquartile range
KCL	King’s College London
LFAT	Lateral flow antigen test
ONS	Office for National Statistics (UK)
PCR	Polymerase chain reaction
SARS-CoV-2	Severe acute respiratory syndrome-related coronavirus 2
UK	United Kingdom of Great Britain and Northern Ireland
USA	United States of America
WHO	World Health Organization

## References

[1] Forni D., Cagliani R., Pontremoli C., Clerici M., Sironi M. (2021). The Substitution Spectra of Coronavirus Genomes. Brief. Bioinform..

[2] Forni D., Cagliani R., Pontremoli C., Mozzi A., Pozzoli U., Clerici M., Sironi M. (2021). Antigenic Variation of SARS-CoV-2 in Response to Immune Pressure. Mol. Ecol..

[3] Anastassopoulou C., Gkizarioti Z., Patrinos G.P., Tsakris A. (2020). Human Genetic Factors Associated with Susceptibility to SARS-CoV-2 Infection and COVID-19 Disease Severity. Hum. Genom..

[4] Fan W., Mar K.B., Sari L., Gaszek I.K., Cheng Q., Evers B.M., Shelton J.M., Wight-Carter M., Siegwart D.J., Lin M.M. (2021). TRIM7 Inhibits Enterovirus Replication and Promotes Emergence of a Viral Variant with Increased Pathogenicity. Cell.

[5] Chen H.L., Lin S.R., Liu H.F., King C.C., Hsieh S.C., Wang W.K. (2008). Evolution of Dengue Virus Type 2 during Two Consecutive Outbreaks with an Increase in Severity in Southern Taiwan in 2001–2002. Am. J. Trop. Med. Hyg..

[6] dos Santos W.G. (2021). Impact of Virus Genetic Variability and Host Immunity for the Success of COVID-19 Vaccines. Biomed. Pharmacother..

[7] Planas D., Veyer D., Baidaliuk A., Staropoli I., Guivel-Benhassine F., Rajah M.M., Planchais C., Porrot F., Robillard N., Puech J. (2021). Reduced Sensitivity of SARS-CoV-2 Variant Delta to Antibody Neutralization. Nature.

[8] Luo C.H., Morris C.P., Sachithanandham J., Amadi A., Gaston D., Li M., Swanson N.J., Schwartz M., Klein E.Y., Pekosz A. (2021). Infection with the SARS-CoV-2 Delta Variant Is Associated with Higher Infectious Virus Loads Compared to the Alpha Variant in Both Unvaccinated and Vaccinated Individuals. medRxiv.

[9] Twohig K.A., Nyberg T., Zaidi A., Thelwall S., Sinnathamby M.A., Aliabadi S., Seaman S.R., Harris R.J., Hope R., Lopez-Bernal J. (2021). Hospital Admission and Emergency Care Attendance Risk for SARS-CoV-2 Delta (B.1.617.2) Compared with Alpha (B.1.1.7) Variants of Concern: A Cohort Study. Lancet Infect. Dis..

[10] Ong S.W.X., Chiew C.J., Ang L.W., Mak T.-M., Cui L., Toh M.P.H.S., Lim Y.D., Lee P.H., Lee T.H., Chia P.Y. (2021). Clinical and Virological Features of SARS-CoV-2 Variants of Concern: A Retrospective Cohort Study Comparing B.1.1.7 (Alpha), B.1.315 (Beta), and B.1.617.2 (Delta). Clin. Infect. Dis..

[11] Lopez Bernal J., Andrews N., Gower C., Gallagher E., Simmons R., Thelwall S., Stowe J., Tessier E., Groves N., Dabrera G. (2021). Effectiveness of Covid-19 Vaccines against the B.1.617.2 (Delta) Variant. N. Engl. J. Med..

[12] Antonelli M., Penfold R.S., Merino J., Sudre C.H., Molteni E., Berry S., Canas L.S., Graham M.S., Klaser K., Modat M. (2021). Risk Factors and Disease Profile of Post-Vaccination SARS-CoV-2 Infection in UK Users of the COVID Symptom Study App: A Prospective, Community-Based, Nested, Case-Control Study. Lancet Infect. Dis..

[13] Molteni E., Sudre C.H., Canas L.S., Bhopal S.S., Hughes R.C., Antonelli M., Murray B., Kläser K., Kerfoot E., Chen L. (2021). Illness Duration and Symptom Profile in Symptomatic UK School-Aged Children Tested for SARS-CoV-2. Lancet Child Adolesc. Health.

[14] Struyf T., Deeks J.J., Dinnes J., Takwoingi Y., Davenport C., Leeflang M.M.G., Spijker R., Hooft L., Emperador D., Domen J. (2021). Signs and Symptoms to Determine If a Patient Presenting in Primary Care or Hospital Outpatient Settings Has COVID-19. Cochrane Database Syst. Rev..

[15] Sudre C., Murray B., Varsavsky T., Graham M., Penfold R., Bowyer R., Pujol J., Klaser K., Antonelli M., Canas L. (2021). Attributes and Predictors of Long COVID. Nat. Med..

[16] Benjamini Y., Hochberg Y. (1995). Controlling the False Discovery Rate: A Practical and Powerful Approach to Multiple Testing. J. R. Stat. Soc. Ser. B.

[17] Galván Casas C., Català A., Carretero Hernández G., Rodríguez-Jiménez P., Fernández-Nieto D., Rodríguez-Villa Lario A., Navarro Fernández I., Ruiz-Villaverde R., Falkenhain-López D., Llamas Velasco M. (2020). Classification of the Cutaneous Manifestations of COVID-19: A Rapid Prospective Nationwide Consensus Study in Spain with 375 Cases. Br. J. Dermatol..

[18] Centers for Disease Control and Prevention (2021). Hospitalizations Associated with COVID-19 Among Children and Adolescents—COVID-NET, 14 States, March 1, 2020–August 14, 2021. Morb. Mortal. Wkly. Rep..

[19] Sheikh A., McMenamin J., Taylor B., Robertson C. (2021). SARS-CoV-2 Delta VOC in Scotland: Demographics, Risk of Hospital Admission, and Vaccine Effectiveness. Lancet.

[20] Veneti L., Salamanca B.V., Seppälä E., Starrfelt J., Storm M.L., Bragstad K., Hungnes O., Bøås H., Kvåle R., Vold L. (2022). No Difference in Risk of Hospitalisation between Reported Cases of the SARS-CoV-2 Delta Variant and Alpha Variant in Norway. Int. J. Infect. Dis..

[21] Kim T.Y., Kim E.C., Agudelo A.Z., Friedman L. (2021). COVID-19 Hospitalization Rate in Children across a Private Hospital Network in the United States: COVID-19 Hospitalization Rate in Children. Arch. Pediatr..

[22] Canas L.S., Österdahl M.F., Deng J., Hu C., Selvachandran S., Polidori L., May A., Molteni E., Murray B., Chen L. (2021). Disentangling Post-Vaccination Symptoms from Early COVID-19. EClinicalMedicine.

[23] Molteni E., Sudre C.H., Canas L.S., Bhopal S.S., Hughes MPH MB ChB R.C., Chen L., Deng J., Murray B., Kerfoot E., Antonelli M. (2021). Illness Characteristics of COVID-19 in Children Infected with the SARS-CoV-2 Delta Variant. medRxiv.

[24] Canas L.S., Sudre C.H., Capdevila Pujol J., Polidori L., Murray B., Molteni E., Graham M.S., Klaser K., Antonelli M., Berry S. (2021). Early Detection of COVID-19 in the UK Using Self-Reported Symptoms: A Large-Scale, Prospective, Epidemiological Surveillance Study. Lancet Digit. Health.

[25] Howard L.M., Garguilo K., Gillon J., LeBlanc K., Seegmiller A.C., Schmitz J.E., Byrne D.W., Domenico H.J., Moore R.P., Webber S.A. (2021). The First 1000 Symptomatic Pediatric SARS-CoV-2 Infections in an Integrated Health Care System: A Prospective Cohort Study. BMC Pediatr..

[26] Thelwall S., Aiano F., Harman K., Dabrera G., Ladhani S.N. (2022). Risk of Hospitalisation and Death in Children with SARS-CoV-2 Delta (B.1.612.2) Infection. Lancet Child Adolesc. Health.

[27] Varsavsky T., Graham M.S., Canas L.S., Ganesh S., Capdevila Pujol J., Sudre C.H., Murray B., Modat M., Jorge Cardoso M., Astley C.M. (2021). Detecting COVID-19 Infection Hotspots in England Using Large-Scale Self-Reported Data from a Mobile Application: A Prospective, Observational Study. Lancet Public Health.

