# Supplementary material for: Illness Characteristics of COVID-19 in Children Infected with the SARS-CoV-2 Delta Variant
Source: Children (Basel). 2022 May 3;9(5):652. doi: 10.3390/children9050652 (PMC9140086; doi:10.3390/children9050652)

SUPPLEMENTARY DOCUMENT
Illness characteristics of COVID-19 in children infected with the SARS-CoV-2 Delta variant

Erika Molteni, Carole H. Sudre, Liane S. Canas, Sunil S. Bhopal, Robert C. Hughes, Liyuan Chen, Jie Deng, Benjamin Murray, Eric Kerfoot, Michela Antonelli, Mark Graham, Kerstin Kläser, Anna May, Christina Hu, Joan Capdevila Pujol, Jonathan Wolf, Prof Alexander Hammers, Prof Tim D. Spector, Prof Sebastien Ourselin, Marc Modat, Claire J. Steves, Michael Absoud, Prof Emma L. Duncan.

Supplementary Methods.

The COVID Symptom Study (administered through the ZOE COVID Study App) was launched jointly by ZOE Limited and King's College London on March 24, 2020. Data were acquired through a mobile application.[25] At time of registration through the app, all participants provide consent for their data to be used for research. Participants can withdraw from the study at any time, with their data, (including proxy-reported data) subsequently excluded from analysis.

The app was tested both before launch and before version updates. The app usage workflow was described in the earliest publication about the COVID Symptom Study (doi: 10.1126/science.abc0473). The team of app developers monitor the statistics of app usage and users' input daily, to assess for faults (e.g., problems in the app downloads or logging). No major disruptions were detected during this study.

Conjointly to the app development, software for data extraction, curation and analytics was developed, which guarantees the repeatability of the data curation over time and the consistency on different timestamps, according to stringent predefined criteria (filters). The software, based on Python language, is called ExeTera; documentation and associated code can be downloaded here: https://arxiv.org/abs/2011.00867

Currently 92% of UK adults own a smartphone, with little difference according to socioeconomic status (https://www.statista.com/statistics/300384/mobile-phone-usage-in-the-uk-by-socio-economic-group/).

Supplementary Table S1. Incidence (%) of the Alpha and Delta variants of SARS-CoV-2 in United Kingdom, in two timeframes: December 28, 2020 to May 6, 2021, and May 26, 2021 to July 8, 2021 (https://assets.publishing.service.gov.uk/government/uploads/system/uploads/attachment_data/file/975754/Variants_of_Concern_Technical_Briefing_8_Data_England.xlsx; GISAID https://covariants.org).

Week of	Alpha (B.1.1.7)	Delta (B.1.617.2)
30 December 2020	78·1	0
6 January	82·3	0
13 January	87·6	0
20 January	90·8	0
27 January	93·8	0
3 February	95·7	0
10 February	97·4	0
17 February	98·1	0
24 February	98·6	0
3 March	98·8	0
10 March	98·9	0
17 March	>99	0
24 March	98	0
31 March	98	0
7 April	95	2
14 April	92	6
21 April	87	9
28 April	76	24
5 May	71·5	25·8
26 May	19·3	80·0
2 June	9·4	90·2
9 June	5	95
16 June	2	98
23 June	2	98
30 June	<1	>99
7 July	<1	>99

Supplementary Table S2. List of symptom questions asked by the ZOE COVID Study app during the study period.

**Symptom**	**COVID Symptom Study app question**
Fever	Fever (at least 37.8C or 100F)
Persistent Cough	Persistent cough (coughing a lot for more than an hour or 3 or more coughing episodes in 24 hours)
Fatigue	Unusual fatigue (no; mild fatigue; severe fatigue/ I struggle to get out of bed)
Dyspnoea	Shortness of breath or trouble breathing (no; yes mild symptoms/ slight shortness of breath during ordinary activity: yes significant symptoms/ breathing is comfortable only at rest; yes, severe symptoms/ breathing is difficult even at rest).
Anosmia/Ageusia	Loss of smell / taste
Hoarse Voice	Unusually hoarse voice
Chest Pain	Unusual chest pain or tightness in your chest
Abdominal Pain	Unusual abdominal pain or stomach-ache
Diarrhoea	Diarrhoea
Stools	How many loose stools in the last 24 hours?
Confusion	Confusion, disorientation or drowsiness
Eye Soreness	Do your eyes have any unusual eye-soreness or discomfort (e.g. light sensitivity, excessive tears, or pink/red eye)?

Loss of appetite	Skipping meals
Headache	Headache
Nausea	Nausea or vomiting
Dizziness	Dizziness or light-headedness
Sore Throat	Sore or painful throat
Myalgias	Unusual strong muscle pains or aches
Red Welts	Raised, red, itchy welts on the skin or sudden swelling of the face or lips
Blisters	Red/purple sores or blisters on your feet, including your toes
Allergy Exacerbation	Increase in your usual allergy symptoms
Rashes	Rash on your arms or torso
Sensitive Skin	Strange, unpleasant sensations in your skin like pins & needles or burning
Hair Loss	Unusual hair loss
Low Mood	Feeling down, depressed, or hopeless
Brain Fog	Loss of concentration or memory (brain fog)
Dysosmia/Dysgeusia	Altered smell / taste (things smell or taste different to usual)
Rhinorrhoea	Runny nose
Sneezing	Sneezing more than usual
Ear Pain	Earache

Tinnitus	Ringing in your ears
Lymphadenopathy	Swollen neck glands
Palpitations	Unusually fast or irregular heartbeat (palpitations)
Arthralgias	Unusual joint pains or aches
Mouth Ulcers	Mouth or tongue ulcers
Tongue Changes	Changes to tongue surface

Supplementary Table S3. Symptoms over the entire illness duration in children testing positive for Alpha and Delta variants of SARS-CoV-2. Data refers to younger (age 5–11 years) and older (age 12-17 years) children. Only children whose illness duration was <28 days are included. Symptom onset between December 28, 2020 and May 6, 2021 was attributed to infection with the Alpha variant, and symptom onset between May 26, 2021 and July 8, 2021 to infection with the Delta variant.

	Younger children		Older children		All children	
	Alpha (n=276)	Delta (n=227)	Alpha (n=418)	Delta (n=479)	Alpha (n=694)	Delta (n=706)
Headache	110 (39·9)	138 (60·8)	257 (61·5)	353 (73·7)	367 (52·9)	491 (69·5)
Fatigue	107 (38·8)	111 (48·9)	238 (56·9)	288 (60·1)	345 (49·7)	399 (56·5)
Rhinorrhoea	130 (47·1)	122 (53·7)	198 (47·4)	276 (57·6)	328 (47·3)	398 (56·4)
Sore throat	90 (32·6)	83 (36·6)	191 (45·7)	290 (60·5)	281 (40·5)	373 (52·8)
Sneezing	96 (34·8)	82 (36·1)	131 (31·3)	167 (34·9)	227 (32·7)	249 (35·3)
Anosmia/Dysosmia	41 (14·9)	45 (19·8)	167 (40·0)	224 (46·8)	208 (30·0)	269 (38·1)
Fever	88 (31·9)	105 (46·3)	113 (27·0)	207 (43·2)	201 (29·0)	312 (44·2)
Persistent cough	74 (26·8)	60 (26·4)	108 (25·8)	148 (30·9)	182 (26·2)	208 (29·5)
Loss of appetite	46 (16·7)	43 (18·9)	80 (19·1)	122 (25·5)	126 (18·2)	165 (23·4)
Abdominal pain	53 (19·2)	48 (21·1)	66 (15·8)	65 (13·6)	119 (17·1)	113 (16·0)
Dizziness	26 (9·4)	25 (11·0)	86 (20·6)	153 (31·9)	112 (16·1)	178 (25·2)
Chills or shivers	27 (9·8)	41 (18·1)	78 (18·7)	114 (23·8)	105 (15·1)	155 (22·0)
Myalgias	18 (6·5)	14 (6·2)	77 (18·4)	80 (16·7)	95 (13·7)	94 (13·3)
Eye soreness	19 (6·9)	34 (15·0)	74 (17·7)	107 (22·3)	93 (13·4)	141 (20·0)
Nausea	26 (9·4)	30 (13·2)	60 (14·4)	80 (16·7)	86 (12·4)	110 (15·6)
Hoarse voice	31 (11·2)	37 (16·3)	53 (12·7)	98 (20·5)	84 (12·1)	135 (19·1)
Confusion	9 (3·3)	9 (4·0)	42 (10·0)	56 (11·7)	51 (7·3)	65 (9·2)

Diarrhoea	20 (7·2)	18 (7·9)	27 (6·5)	36 (7·5)	47 (6·8)	54 (7·6)
Dyspnoea	9 (3·3)	6 (2·6)	37 (8·9)	50 (10·4)	46 (6·6)	56 (7.9)
Low mood	15 (5·4)	7 (3·1)	29 (6·9)	33 (6·9)	44 (6·3)	40 (5·7)
Chest pain	11 (4·0)	7 (3·1)	32 (7·7)	43 (9·0)	43 (6·2)	50 (7·1)
Lymphadenopathy	12 (4·3)	12 (5·3)	30 (7·2)	38 (7·9)	42 (6·1)	50 (7·1)
Earache	11 (4·0)	14 (6·2)	24 (5·7)	41 (8·6)	35 (5·0)	55 (7·8)
Rash	15 (5·4)	9 (4·0)	13 (3·1)	19 (4·0)	28 (4·0)	28 (4·0)
Delirium	2 (0·7)	5 (2·2)	23 (5·5)	38 (7·9)	25 (3·6)	43 (6·1)
Tinnitus	6 (2·2)	7 (3·1)	16 (3·8)	21 (4·4)	22 (3·2)	28 (4·0)
Sensitive skin	6 (2·2)	7 (3·1)	14 (3·3)	22 (4·6)	20 (2·9)	29 (4·1)
Red welts on face or lips	8 (2·9)	5 (2·2)	9 (2·2)	10 (2·1)	17 (2·4)	15 (2·1)
Palpitations	4 (1·4)	6 (2·6)	10 (2·4)	7 (1·5)	14 (2·0)	13 (1·8)
Blisters	1 (0·4)	1 (0·4)	5 (1·2)	5 (1·0)	6 (0·9)	6 (0·8)
Hair loss	0 (0·0)	0 (0·0)	1 (0·2)	2 (0·4)	1 (0·1)	2 (0·3)

Supplementary Table S4. Odds ratios for symptoms presenting with SARS-CoV-2 infection, comparing Delta to Alpha infection. Odds ratios are adjusted for age and sex. Only children with illness duration <28 days are included. The bold font and asterisk indicate statistical significance after FDR correction with α>0.05. Symptoms are ranked based on prevalence in the overall cohort during the period of Alpha predominance.

	Younger children		Older children		All children	
	Odd Ratio (CI)	p-value	Odd Ratio (CI)	p-value	Odd Ratio (CI)	p-value
Headache	**2·31 (1·60-3·33)**	**<0·001***	**1·81 (1·36-2·42)**	**<0·001***	**1·97 (1·57-2·46)**	**<0·001***
Fatigue	1·46 (1·02-2·09)	0·039	1·16 (0·89-1·52)	0·280	1·25 (1·01-1·55)	0·038
Rhinorrhoea	1·31 (0·92-1·86)	0·139	**1·52 (1·17-1·98)**	**0·002***	**1·44 (1·17-1·78)**	**0·001***
Sore throat	1·13 (0·77-1·64)	0·534	**1·87 (1·43-2·45)**	**<0·001***	**1·58 (1·27-1·97)**	**<0·001***
Sneezing	1·09 (0·75-1·57)	0·662	1·17 (0·88-1·54)	0·278	1·14 (0·91-1·42)	0·255
Anosmia/Dysosmia	1·29 (0·80-2·09)	0·292	1·36 (1·04-1·78)	0·025	**1·36 (1·08-1·72)**	**0·010***
Fever	**1·89 (1·31-2·73)**	**0·001***	**2·05 (1·55-2·72)**	**<0·001***	**1·99 (1·59-2·49)**	**<0·001***
Persistent cough	1·02 (0·68-1·51)	0·942	1·29 (0·96-1·72)	0·093	1·17 (0·92-1·48)	0·193
Loss of appetite	1·18 (0·74-1·87)	0·486	1·46 (1·06-2·01)	0·02	1·35 (1·04-1·75)	0·024
Abdominal pain	1·16 (0·75-1·81)	0·503	0·83 (0·57-1·21)	0·342	0·96 (0·72-1·27)	0·752
Dizziness	1·12 (0·62-2·02)	0·717	**1·86 (1·37-2·54)**	**<0·001***	**1·69 (1·29-2·21)**	**<0·001***
Chills or shivers	1·97 (1·17-3·33)	0·011	1·38 (1-1·91)	0·053	**1·53 (1·16-2·01)**	**0·003***
Myalgias	0·91 (0·44-1·87)	0·788	0·9 (0·64-1·27)	0·554	0·91 (0·66-1·24)	0·540
Eye soreness	**2·40 (1·32-4·38)**	**0·004***	1·34 (0·97-1·87)	0·080	**1·55 (1·16-2·07)**	**0·003***
Nausea	1·43 (0·81-2·50)	0·218	1·21 (0·84-1·74)	0·315	1·27 (0·94-1·73)	0·122
Hoarse voice	1·58 (0·94-2·65)	0·083	**1·78 (1·24-2·57)**	**0·002***	**1·70 (1·26-2·28)**	**<0·001***
Confusion	1·17 (0·45-3·01)	0·749	1·2 (0·79-1·84)	0·396	1·21 (0·82-1·78)	0·328
Diarrhoea	1·21 (0·62-2·37)	0·578	1·16 (0·69-1·95)	0·567	1·17 (0·78-1·76)	0·451
Dyspnoea	0·77 (0·27-2·22)	0·632	1·23 (0·79-1·93)	0·366	1·15 (0·76-1·73)	0·513
Low mood	0·52 (0·21-1·32)	0·170	1·01 (0·6-1·7)	0·972	0·85 (0·55-1·33)	0·484
Chest pain	0·77 (0·29-2·04)	0·604	1·22 (0·75-1·97)	0·421	1·10 (0·72-1·68)	0·670
Lymphadenopathy	1·22 (0·53-2·78)	0·640	1·13 (0·69-1·86)	0·635	1·15 (0·75-1·76)	0·525
Earache	1·58 (0·70-3·58)	0·267	1·58 (0·94-2·67)	0·087	1·56 (1·00-2·42)	0·048

Rash	0·67 (0·29-1·58)	0·361	1·29 (0·63-2·65)	0·485	0·98 (0·58-1·68)	0·951
Delirium	2·94 (0·56-15·38)	0·202	1·48 (0·87-2·53)	0·153	1·64 (0·98-2·72)	0·058
Tinnitus	1·44 (0·47-4·43)	0·529	1·16 (0·59-2·26)	0·667	1·23 (0·70-2·17)	0·477
Sensitive skin	1·39 (0·46-4·22)	0·560	1·38 (0·7-2·74)	0·352	1·41 (0·79-2·52)	0·244
Palpitations	1·94 (0·54-7·01)	0·313	0·62 (0·23-1·64)	0·333	0·89 (0·42-1·92)	0·773
Red welts on face or lips	0·75 (0·24-2·35)	0·625	0·98 (0·39-2·43)	0·962	0·88 (0·43-1·78)	0·717
Blisters	1·11 (0·07-18·16)	0·942	0·88 (0·25-3·06)	0·838	0·92 (0·29-2·87)	0·886
Hair loss	0·00 (0·00-0·00)	-	2·15 (0·19-24·29)	0·536	2·15 (0·19-24·29)	0·535

*Statistically significant after FDR correction for multiple tests at 0·05

Supplementary Table S5. Symptom presentation over the first 28 days of the disease in children testing negative for SARS-CoV-2. Data refers to younger (age 5–11 years) and older (age 12-17 years) children, and the cohort overall. Individuals were grouped by symptom onset date, analogous to the positively-tested children (Alpha: between December 28, 2020 and May 6, 2021; Delta: between May 26, 2021 and July 8, 2021).

	Younger children		Older children		All children	
	Alpha (n=3,748)	Delta (n=1,117)	Alpha (n=2,285)	Delta (n=231)	Alpha (n=6,033)	Delta (n=1,348)
Headache	1,025 (27.3)	255 (22·8)	1,026 (44·9)	98 (42·4)	2,051 (34·0)	353 (26·2)
Fatigue	937 (25·0)	189 (16·9)	751 (32·9)	63 (27·3)	1,688 (28·0)	252 (18·7)
Rhinorrhoea	1,902 (50·7)	673 (60·3)	596 (26·1)	123 (53·2)	2,498 (41·4)	796 (59·1)
Sore throat	1,496 (39·9)	497 (44·5)	730 (31·9)	130 (56·3)	2,226 (36·9)	627 (46·5)
Sneezing	1,210 (32·3)	391 (35·0)	415 (18·2)	89 (38·5)	1,625 (26·9)	480 (35·6)
Anosmia/Dysosmia	188 (5·0)	33 (3·0)	160 (7·0)	16 (6·9)	348 (5·8)	49 (3·6)
Fever	1,212 (32·3)	302 (27·0)	484 (21·2)	48 (20·8)	1,696 (28·1)	350 (26·0)
Persistent cough	932 (24·9)	422 (37·8)	224 (9·8)	63 (27·3)	1,156 (19·2)	485 (36·0)
Loss of appetite	424 (11·3)	97 (8·7)	303 (13·3)	16 (6·9)	727 (12·1)	113 (8·4)
Abdominal pain	753 (20·1)	142 (12·7)	485 (21·2)	30 (13·0)	1,238 (20·5)	172 (12·8)
Dizziness	193 (5·1)	32 (2·9)	344 (15·1)	32 (13·9)	537 (8·9)	64 (4·7)
Chills or shivers	432 (11·5)	89 (8·0)	331 (14·5)	20 (8·7)	763 (12·6)	109 (8·1)
Myalgias	132 (3·5)	23 (2·1)	167 (7·3)	12 (5·2)	299 (5·0)	35 (2·6)
Eye soreness	215 (5·7)	77 (6·9)	209 (9·1)	22 (9·5)	424 (7·0)	99 (7·3)
Nausea	484 (12·9)	106 (9·5)	511 (22·4)	28 (12·1)	995 (16·5)	134 (9·9)
Hoarse voice	452 (12·1)	166 (14·9)	139 (6·1)	30 (13·0)	591 (9·8)	196 (14·5)
Confusion	63 (1·7)	6 (0·5)	129 (5·6)	9 (3·9)	192 (3·2)	15 (1·1)
Diarrhoea	258 (6·9)	62 (5·6)	210 (9·2)	13 (5·6)	468 (7·8)	75 (5·6)
Dyspnoea	101 (2·7)	39 (3·5)	99 (4·3)	10 (4·3)	200 (3·3)	49 (3·6)
Low mood	104 (2·8)	11 (1·0)	190 (8·3)	7 (3·0)	294 (4·9)	18 (1·3)

Chest pain	71 (1·9)	19 (1·7)	114 (5·0)	12 (5·2)	185 (3·1)	31 (2·3)
Lymphadenopathy	236 (6·3)	38 (3·4)	159 (7·0)	21 (9·1)	395 (6·5)	59 (4·4)
Earache	154 (4·1)	50 (4·5)	132 (5·8)	14 (6·1)	286 (4·7)	64 (4·7)
Rash	148 (3·9)	23 (2·1)	97 (4·2)	3 (1·3)	245 (4·1)	26 (1·9)
Delirium	48 (1·3)	13 (1·2)	70 (3·1)	3 (1·3)	118 (2·0)	16 (1·2)
Tinnitus	38 (1·0)	4 (0·4)	64 (2·8)	4 (1·7)	102 (1·7)	8 (0·6)
Sensitive skin	38 (1·0)	4 (0·4)	64 (2·8)	1 (0·4)	102 (1·7)	5 (0·4)
Palpitations	18 (0·5)	4 (0·4)	45 (2·0)	1 (0·4)	63 (1·0)	5 (0·4)
Red welts on face or lips	89 (2·4)	11 (1·0)	58 (2·5)	5 (2·2)	147 (2·4)	16 (1·2)
Blisters	48 (1·3)	2 (0·2)	106 (4·6)	0 (0·0)	154 (2·6)	2 (0·1)
Hair loss	0 (0·0)	0 (0·0)	7 (0·3)	1 (0·4)	7 (0·1)	1 (0·1)

Supplementary Figure S1. Prevalence of symptoms reported by children testing negative for COVID-19. Data are reported in younger (aged 5–11 years) and older (aged 12–17 years) children, for symptoms presenting within 28 days, during periods of Alpha and Delta SARS-CoV-2 variant predominance.

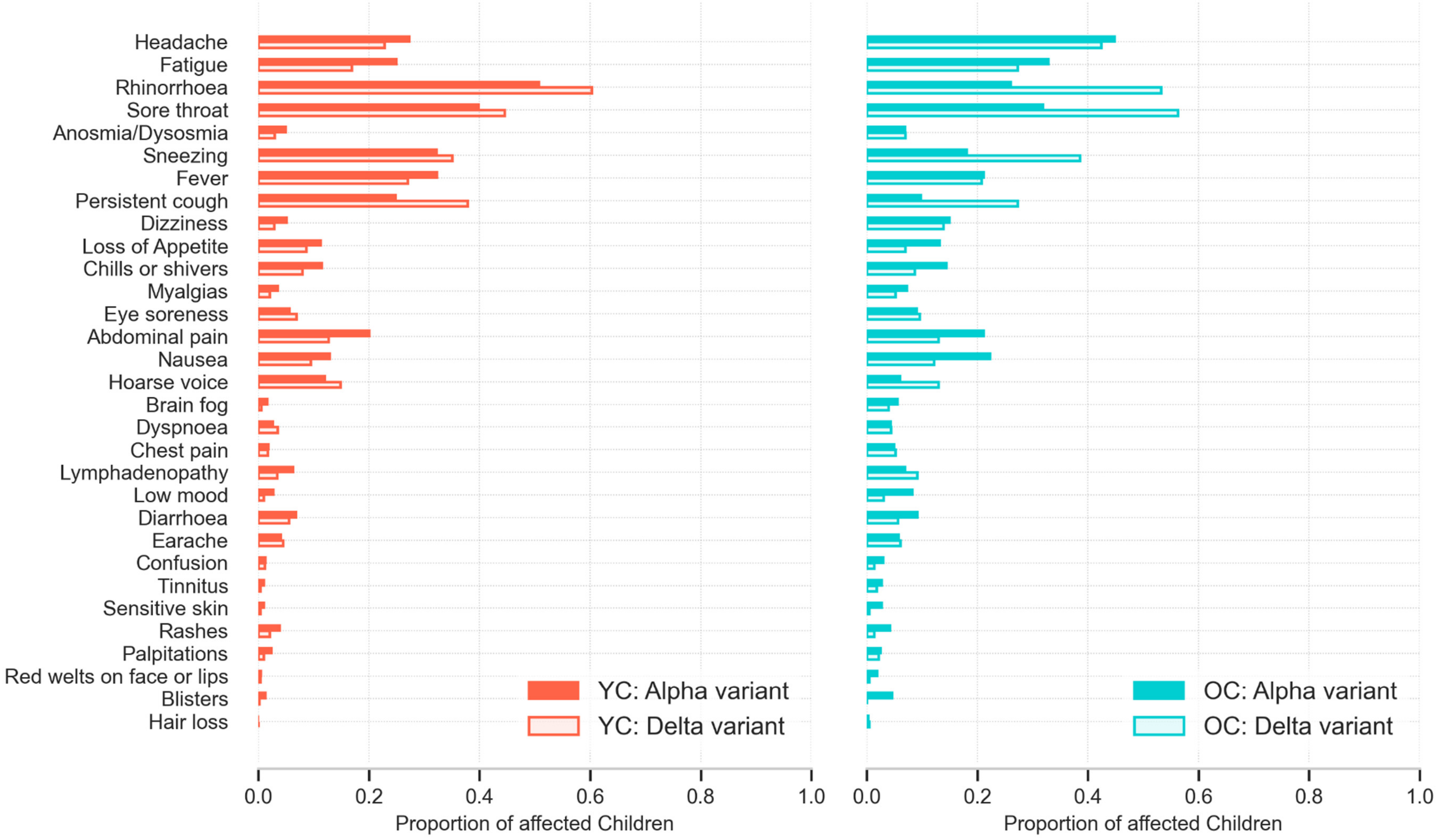

Supplement: Supplementary file 1 [file children-09-00652-s001.zip › children-1690954-supplementary.pdf]
